# Supplementary material for: Mechanosensitive Differentiation of Human iPS Cell-Derived Podocytes
Source: Bioengineering (Basel). 2024 Oct 17;11(10):1038. doi: 10.3390/bioengineering11101038 (PMC11504473; doi:10.3390/bioengineering11101038)
Supplement: Supplementary file 1 [file bioengineering-11-01038-s001.zip › bioengineering-3234036-supplementary.pdf]

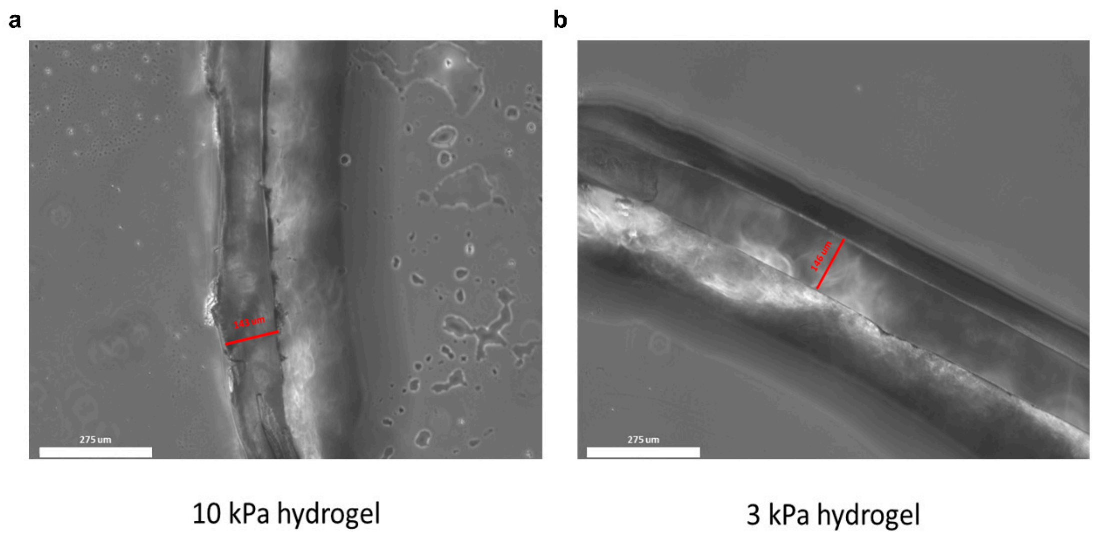

Supplementary Figure S1. Swelling capacity of the hydrogel at equilibrium. (a) Cross section of 10 kPa hydrogel stored in PBS for 10 months; thickness of the hydrogel marked by a red line; scale bar, 275 µm. (b) Cross section of 3 kPa hydrogel stored in PBS for 10 months; thickness of the hydrogel marked by a red line; scale bar, 275 µm.

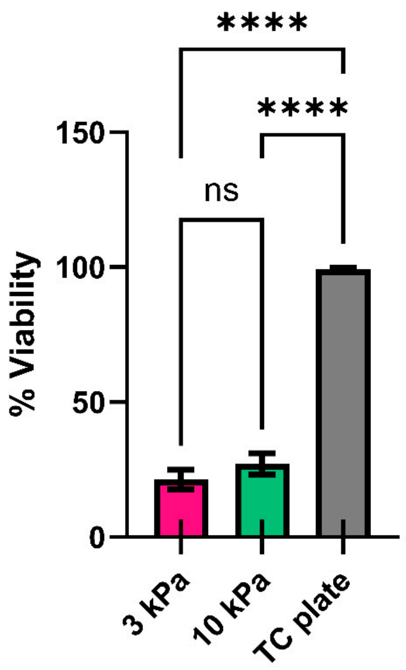

Supplementary Figure S2. Metabolic activity of human iPS cell-derived podocytes differentiated on hydrogels and TC plates. Comparison of the percentage of viable cells on hydrogels of different rigidities and on TC plates. Data were acquired using CCK-8 assay at an absorbance of 450 nm for each sample (N=5, n=24). Data are expressed as mean ± SEM. (ns, not significant; ****p < 0.001).

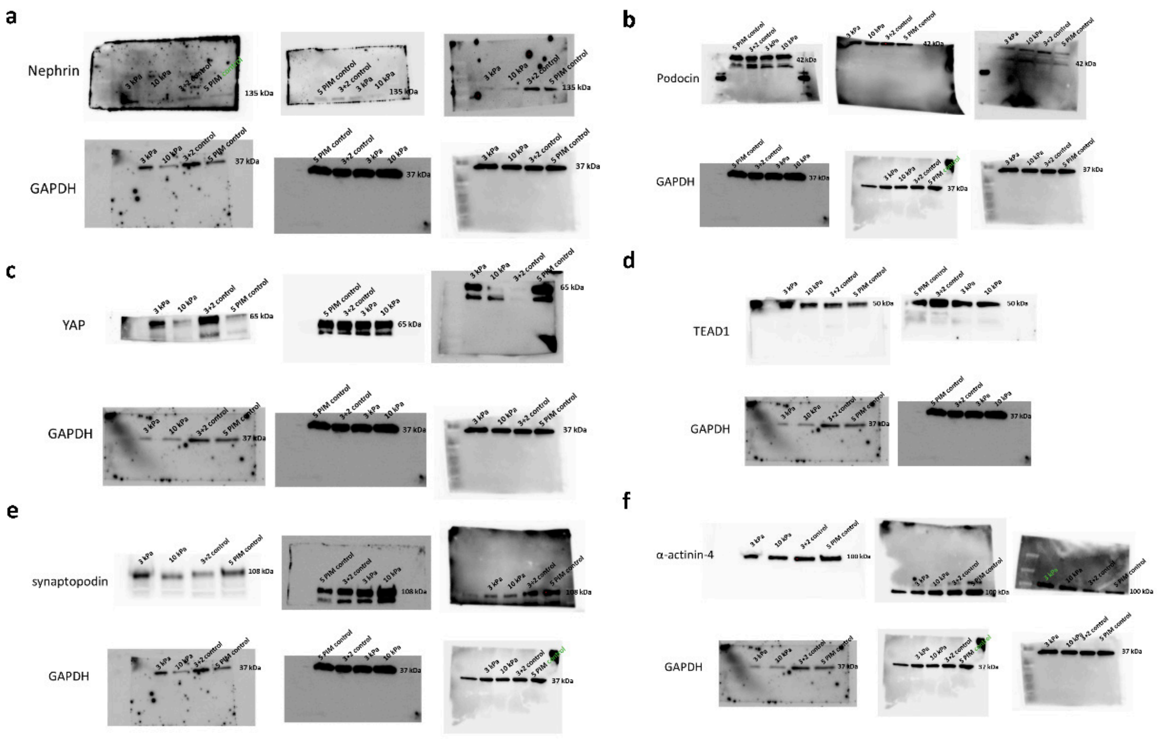

Supplementary Figure S3. Images of the immunoblots used for protein quantification analyses. Representative immunoblots showing expression levels of (a) nephrin, (b) podocin, (c) YAP, (d) TEAD1, (e) synaptopodin, and (f) α-actinin-4. GAPDH was used as a housekeeping protein.

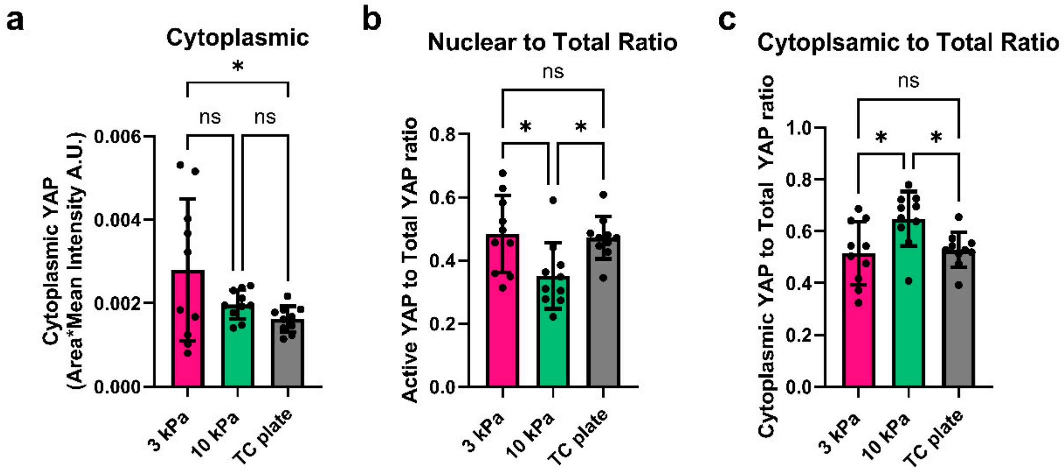

Supplementary Figure S4. YAP Localization in Podocytes Cultured on Hydrogels and TC Plates. (a) Cytoplasmic YAP in cells cultured on each hydrogel and TC plate. (b) Comparative ratios of active nuclear YAP to total YAP levels, and (c) cytoplasmic YAP to total YAP. (N=2, n=10). Data are expressed as mean ± SEM. (ns, not significant; $*p < 0.05$).
